# Missense Variants in the Second Transmembrane Domain of TMEM17 Disrupt Its Stability and Function and Lead to a Wide Phenotypic Spectrum of Ciliopathies

**DOI:** 10.1111/cge.70042

**Published:** 2025-08-21

**Authors:** Lucile Boutaud, Chunmei Li, Candice Moncler, Laure Verlin, Meriem Garfa‐Traoré, Nicolas Bourgon, Dhruvin Akbari, Jeanne Porée, Valentina Serpieri, Marine Panza, Lynda Haddad, Patrick Nitschké, Jacqueline Aziza, Cristina Matt, Enza Maria Valente, Patricia Gargallo, Charlotte Dubucs, Tania Attié‐Bitach, Michel R. Leroux, Sophie Thomas

**Affiliations:** ^1^ INSERM UMR 1163, Institut Imagine Université Paris Cité Paris France; ^2^ Service de Médecine Génomique des Maladies Rares Hôpital Universitaire Necker‐Enfants Malades Paris France; ^3^ Department of Molecular Biology and Biochemistry Simon Fraser University Burnaby British Columbia Canada; ^4^ Centre for Cell Biology, Development, and Disease Simon Fraser University Burnaby British Columbia Canada; ^5^ Cell Imaging Platform, INSERM‐US24‐CNRS UMS 3633 Structure Fédérative de Recherche Necker Paris University Paris France; ^6^ Department of Molecular Medicine University of Pavia Pavia Italy; ^7^ Imagine Institute, Bioinformatics Platform, INSERM UMR 1163 Université de Paris Paris France; ^8^ Département de Pathologie Institut Universitaire du Cancer Toulouse – Oncopole Toulouse France; ^9^ Seccion de Genética Médica del Centro de Educación Médica e Investigaciones Clínicas “Norberto Quirno”, CP (C1431FWO) Buenos Aires Argentina; ^10^ Neurogenetics Research Center, IRCCS, Mondino Foundation Pavia Italy

**Keywords:** ciliopathy, Joubert syndrome, Meckel syndrome, Oro‐Facio‐digital syndrome, primary cilium, TMEM17, transition zone

## Abstract

Ciliopathies are rare genetic disorders characterized by significant genetic and phenotypic variability. Over 140 proteins localized to primary cilia, which are sensory organelles essential for vertebrate development, are implicated. *TMEM17* encodes a transmembrane protein at the ciliary transition zone and was previously proposed as a potential ciliopathy gene, based on reports of individuals from two families with orofaciodigital syndrome type 6 (OFD6) and Joubert syndrome (JS). Here, we report two unrelated fetuses with occipital encephalocele, polydactyly, and kidney cysts, in whom exome sequencing identified a founder homozygous missense variant (Arg94Trp) in TMEM17, affecting a highly conserved residue. This expands the *TMEM17*‐associated phenotypic spectrum to include Meckel syndrome (MKS). Comprehensive functional analyses of all known *TMEM17* variants, using patient tissues/cells and a *C. elegans* model system, demonstrate a loss‐of‐function mechanism. Our study reveals severe functional consequences, including TMEM17 destabilization and mislocalization, anomalies in cilium composition and function, and abrogation of Sonic Hedgehog signaling. These experiments confirm the pathogenicity of all *TMEM17* variants and underscore its essential role at the ciliary transition zone. Collectively, our findings establish *TMEM17* as a *bona fide* ciliopathy gene, associated with a wide phenotypic spectrum ranging from viable syndromes (OFD6 and JS) to a fetal‐lethal condition (MKS).

## Graphical abstract

Missense variants in *TMEM17* disrupt its localization and function at the ciliary transition zone, leading to a wide range of ciliopathy phenotypes, from OFD6 and Joubert syndromes to Meckel syndrome.

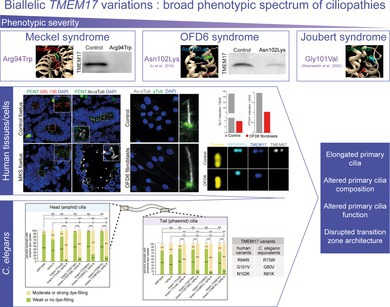

## 1 Introduction

Cilia are evolutionarily conserved, surface‐exposed organelles found across eukaryotes [1, 2, 3, 4]. In animals, two major types exist: motile cilia, which drive movement or fluid flow [5, 6], and primary cilia (PC), immotile structures that function as cellular antennas [7, 8, 9]. PC detect environmental signals and mediate key signaling pathways—such as calcium, cAMP, Hedgehog, and GPCR signaling—essential for vertebrate development and physiology [7, 10, 11, 12, 13, 14, 15].

Rare genetic disorders affecting the formation or function of PC are termed ciliopathies. They are characterized by genetic heterogeneity and overlapping clinical features impacting many organs, especially the brain, retina, kidneys, liver, and skeleton [5, 8, 16, 17, 18, 19]. They also exhibit considerable phenotypic heterogeneity, with most causal genes associated with a broad clinical spectrum ranging from viable single‐organ involvement diseases to fetal‐lethal syndromes [16, 19, 20]. For example, Joubert syndrome (JS, [MIM213300]) presents with a specific midbrain‐hindbrain malformation (molar tooth sign, MTS) and variable organ involvement [21]. Orofaciodigital (OFD) syndromes manifest a co‐occurrence of oral, facial, and digital anomalies and are differentiated through associations with additional features, defining 14 types, including type 6 (OFD6, [MIM 277170]) with additional MTS [22]. At the severe end, Meckel syndrome (MKS, [MIM249000]) is a lethal condition characterized by neural tube closure defect (usually occipital encephalocele), polydactyly, cystic kidneys, and hepatic ductal dysgenesis.

Cilia are built from a microtubule‐based axoneme extending from a modified centriole termed basal body [23, 24]. Intraflagellar transport (IFT) systems maintain ciliary homeostasis by shuttling proteins in and out [25, 26, 27]. At the cilium's base, the transition zone (TZ) acts as a selective barrier or “ciliary gate” [25, 28, 29, 30, 31], characterized by Y‐shaped links between the axoneme and membrane. The TZ contains at least 14 conserved, mostly membrane‐associated proteins, though its gating mechanisms—such as lipid microdomains or size filters—remain incompletely understood [29]. Many TZ genes are implicated in ciliopathies [32, 33, 34, 35, 36, 37, 38]. *TMEM17*, encoding a four‐transmembrane TZ protein conserved across ciliated eukaryotes [39, 40], has been proposed as causal in OFD6 and JS based on biallelic missense variants in two patients [39, 41]. First localized to the TZ by Chih et al. (2011), TMEM17 was among the earliest mammalian proteins linked to ciliary gating [42, 43]. Its *C. elegans* ortholog, *TMEM‐17*, also localizes to the TZ and supports ciliogenesis [44].

Here, we show that fetuses from two unrelated families with characteristic MKS features harbor the same founder homozygous missense variant (NM_198276.3:c.280C>T, Arg94Trp). This conserved variant results in complete loss of TMEM17 protein and severe ciliogenesis defects, as evidenced by immunohistochemistry (IHC) on fetal kidney sections. We also showed that the *TMEM17* variant previously linked to OFD6 impairs TMEM17 stability, leaving only 25% residual protein; although able to localize to the TZ, the mutated protein leads to severe defects in cilium biogenesis and function. Specifically, PC from OFD6 patient cells are longer with altered protein composition and impaired ability to transduce the Sonic Hedgehog (SHH) pathway. We further modeled the Arg94Trp variant in a *C. elegans* model system, as well as the two previously reported variants (Gly101Val and Asn102Lys) with unknown pathological mechanisms [39, 41], and found that all three disrupt TMEM‐17 function. Together, our findings establish *TMEM17* as a *bona fide* ciliopathy gene with a broad phenotypic spectrum and provide evidence that deleterious missense variants in *TMEM17* involve amino acids that reside within a transmembrane “mutational hotspot” crucial for its biological function.

## 2 Materials and Methods

### 2.1 Autopsy

According to French and Argentinian laws, a complete fetal autopsy was performed after parental consent and according to standardized protocols as previously described [45].

### 2.2 Trio Exome Sequencing

Genomic DNA was extracted from fetal tissues (fetus 1 and fetus 2) or peripheral blood samples (parents). Details regarding library preparation, exome capture, and sequencing methods as well as bioinformatics analysis can be found in the Supplementary Methods S1.

### 2.3 Functional Analysis on Human Tissues/Cells

Cell culture, reverse transcription, real‐time PCR, and Western blot were performed as described previously, along with immunofluorescent analyses (IF) on cells and fetal kidney sections [45, 46]. Additional details, including those regarding statistical analysis, are provided in the Supplementary Methods S1. All antibodies used are listed in the Supplementary Table 1. Ultrastructure expansion microscopy (U‐ExM) and image analysis are described in the Supplementary Methods and in the Supplementary Figure 6.

### 2.4 Modeling of *TMEM17* Patient Variants Using *C. elegans* as a Model System

Details regarding *C. elegans* strain creation and maintenance, ciliary protein localization experiments, and ciliary function analyses are detailed in the Supplementary Methods S1.

## 3 Results

### 3.1 Likely Pathogenic TMEM17 Arg94Trp Variant Identified in Two Unrelated Families Presenting With Meckel Syndrome

**FIGURE 1:**
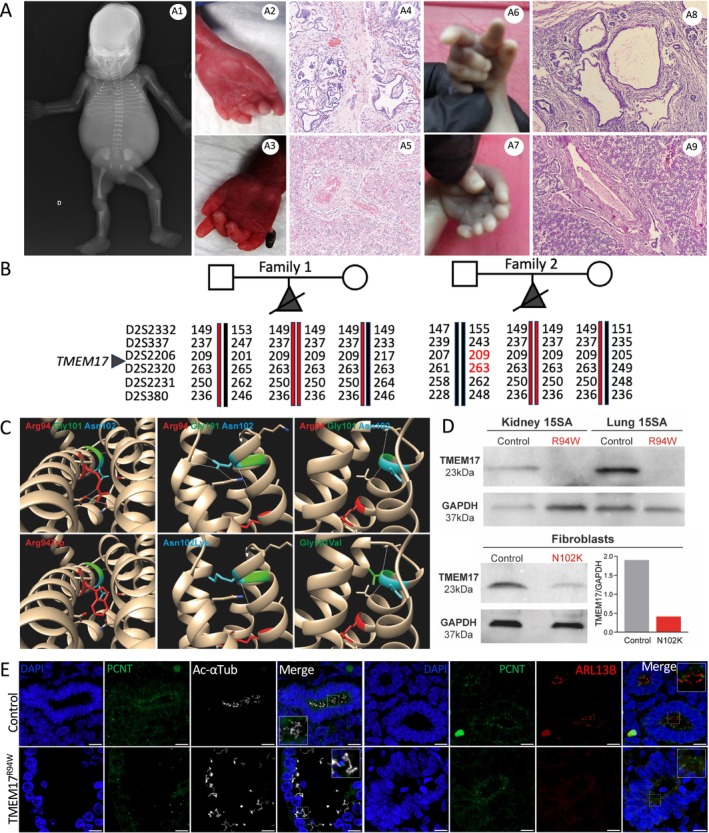
**Phenotypic and functional consequences of a homozygous TMEM17 variant in two unrelated families. (A)** Case 1 (family 1) showed shortened, curved long bones (A1), postaxial polydactyly of hands and feet (A2, A3), enlarged cystic kidneys (A4), and bile duct proliferation (A5). Case 2 (family 2) also displayed features of MKS, including hexadactyly (A6, A7), cystic kidney parenchyma (A8), and liver fibrosis (A9). **(B)** Haplotype analysis using microsatellite markers supports a founder effect with chromosome 2 haplotype shared by the affected fetuses. Family 2 haplotype is consistent with maternal uniparental isodisomy of chromosome 2. **(C)** 3D modeling of TMEM17^R94W^ (MKS, this study), TMEM17^N102K^ (OFD6 [39]), and TMEM17^G101V^ (JS^39^), variants based on AlphaFold prediction of TMEM17 (Uniprot Q86X19) and visualized with UCSF Chimera, shows that all three are destabilizing. R94W disrupts a salt bridge with Glu91; G101V alters a conserved buried glycine; N102K introduces a buried charged residue, disrupting key interactions. **(D)** Western blot of kidney/lung tissues from case 1 (TMEM17^R94W^) and an age‐matched control and from TMEM17^N102K^ and control fibroblasts shows complete loss of TMEM17^R94W^ protein and ~20% residual TMEM17^N102K^, demonstrating that both variants impair protein stability. **(E)** IHC on case 1 (TMEM17^R94W^) kidney sections reveals normal cilia number with elongated axonemes lacking ARL13B, indicating altered ciliary composition. Scale bar: 20 μm.

High‐throughput sequencing (HTS) of a fetal ciliopathy cohort uncovered a homozygous *TMEM17* variant in a fetus from a related European couple (Family 1, Case 1), with features of MKS. Postmortem examination revealed a male fetus with occipital encephalocele, postaxial polydactyly (hands and feet), shortened curved long bones, posterior cleft palate, enlarged polycystic kidneys, and bile duct proliferation (Figure 1A, S1 Supplementary Table 2). The variant (NM_198276.3: c.280C>T, p.(Arg94Trp)) was inherited from heterozygous parents and is reported in gnomAD as only heterozygous at very low frequency (0.00001061; South America: 1/35370, Europe: 2/129084). Arg94 is highly conserved across metazoans, green algae, and protists (S1 Supplementary Figure 1), and the substitution is predicted deleterious by AlphaMissense (0.931), REVEL (0.905), and CADD (Phred 28.6). Structural modeling indicates the Arg94Trp substitution disrupts TMEM17 conformation (Figure 1C). Conservation of this residue in related TZ‐localized proteins TMEM216 (MKS2) and TMEM80 [39] further supports its functional relevance (S1 Supplementary Figure 1).

The same homozygous variant (NM_198276.3: c.280C>T, p.[Arg94Trp]) was identified in a second fetus (Family 2, Case 2) with classic MKS features: microcephaly, occipital encephalocele, polydactyly, enlarged microcystic kidneys, oligohydramnios, spine curvature, and curved lower limbs (Figure 1A, S1 Supplementary Table 2). Sanger sequencing revealed that the mother is heterozygous, but the father lacks the variant (parentage confirmed). Paternal deletion was excluded by HTS (coverage ~300X). Microsatellite analysis confirmed haplotype heterozygosity in the father and argued for a chromosome 2 maternal uniparental isodisomy, which seems complete, as suggested by trio SNP analysis all along chromosome 2. In Family 1, microsatellite analysis revealed shared haplotypes between the two fetuses, supporting a founder effect (Figure 1B).

Remarkably, three additional pathogenic variants in *TMEM216* affect the same conserved arginine residue (Arg73), which is mutated in both MKS and JS (S1 Supplementary Figure 1), suggesting that this position may represent a mutational hotspot. Additionally, two previously reported TMEM17 variants (Gly101Val in JS, Asn102Lys in OFD6) also impact nearby residues highly conserved across TMEM17, TMEM216, and TMEM80 (S1 Supplementary Figure 1).

### 3.2 *TMEM17* Variants Cause Protein Instability and Ciliary Defects

To investigate the pathomechanisms associated with *TMEM17* variants, we assessed their impact at the protein level. 3D modeling of human TMEM17 using AlphaFold structure prediction (AF‐Q86X19) predicted structural damage to all three variants by affecting buried residues critical for protein stability, disrupting salt bridges or hydrogen bonds, or introducing steric hindrance (Figure 1C). Western blot analysis showed undetectable TMEM17 protein in kidney and lung tissues from Case 1 (TMEM17^R94W^) and a 75% reduction in fibroblasts from the OFD6 patient (TMEM17^R94W^) compared to control cells (Figure 1D).

**FIGURE 2:**
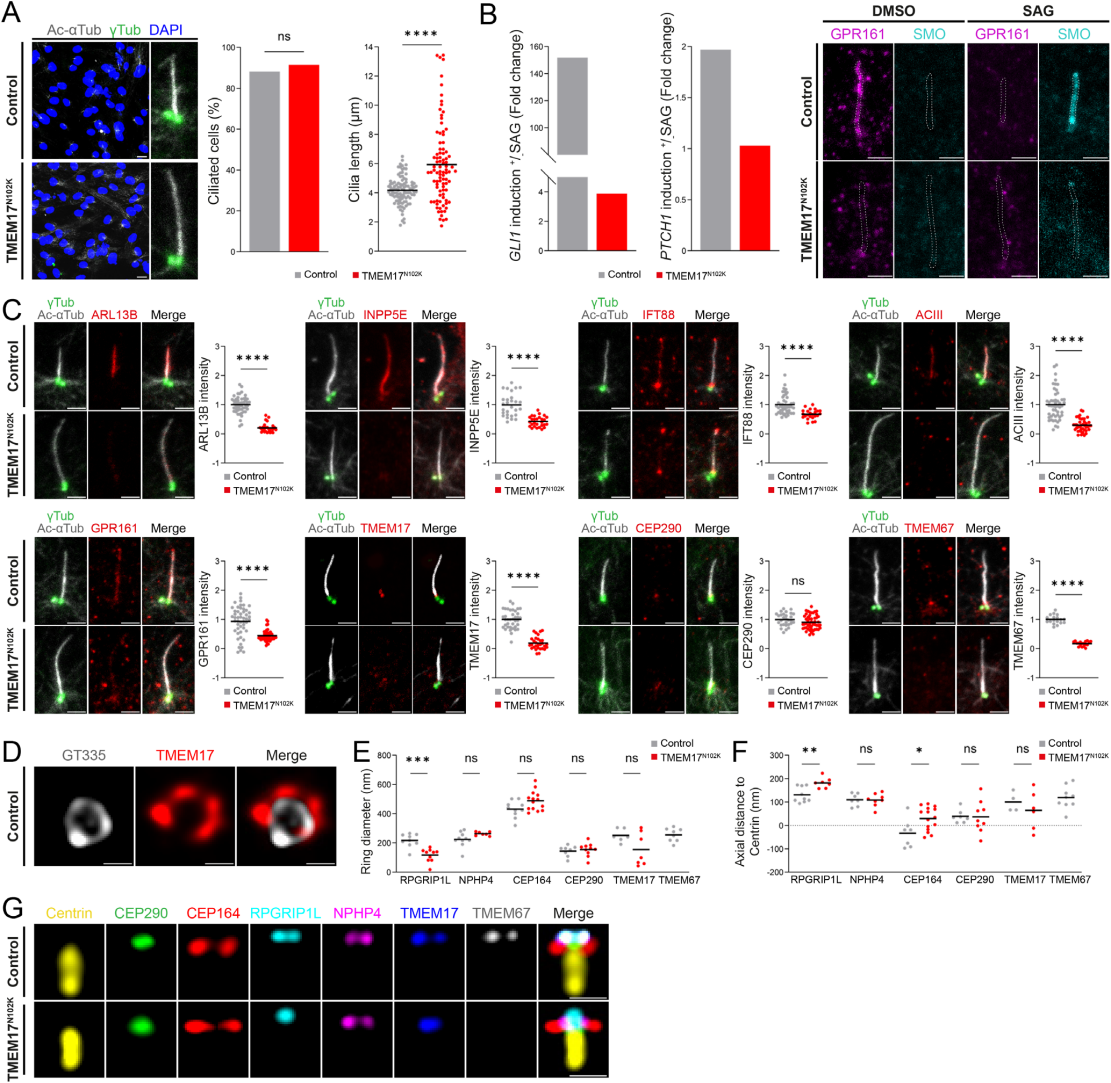
**TMEM17** ^**N102K**^ **impairs PC biogenesis and function. (A)** IF on TMEM17^N102K^ fibroblasts shows normal cilia frequency but significantly elongated axonemes vs. control cells (****p < 0.0001, Mann–Whitney). Scale bar: 20 μm. **(B)** SHH pathway analysis shows impaired induction of *GLI1/PTCH1* and abnormal trafficking of GPR161/SMO in patient vs. control cells upon SAG stimulation. Scale bar: 2 μm. **(C)** IF on TMEM17^N102K^ fibroblasts shows reduced TMEM17 at the TZ and complete absence of TMEM67, while CEP290 remains. Axonemal levels of INPP5E, GPR161, ACIII, ARL13B, and IFT88 are decreased (****p < 0.0001). Scale bar: 2 μm. **(D)** U‐ExM in control fibroblasts shows that TMEM17 forms a concentric, discontinuous ring within the TZ. Scale bar: 0.5 μm. **(E)** Comparison of ring diameters (mean ± SD) of TF/TZ proteins based on radial distribution around the axoneme. In controls, TMEM17 (251 ± 45 nm) aligns with TMEM67 (255 ± 45 nm) at the TZ membrane, while NPHP4 (224 ± 50 nm) and RPGRIP1L (217 ± 46 nm) are positioned closer to the axoneme. In patient cells, TMEM67 is absent and TMEM17 is strongly reduced, precluding measurement; RPGRIP1L appears significantly closer to the axoneme (117 ± 46 nm vs. 217 ± 46 nm in controls). **(F)** Axial measurements in control cells show that all TZ proteins lie ~110 nm distal to centrin, while in patient cells, RPGRIP1L shifts distally (182 ± 23 nm), disrupting normal architecture. **(G)** U‐ExM overlay of TZ proteins reveals disrupted organization in TMEM17^N102K^ fibroblasts, with loss of TMEM67, strong reduction of TMEM17, and mislocalized RPGRIP1L. Scale bar: 0.5 μm. ***p < 0.0005, **p < 0.001, *p < 0.005.

IHC on Case 1 kidney sections, using pericentrin (PCNT) and acetylated‐alpha‐Tubulin (Ac‐αTub) antibodies to mark basal bodies and cilia, revealed abnormal cilium morphology and absence of ARL13B staining, indicating defective trafficking of a ciliary membrane component (Figure 1E). In serum‐starved TMEM17^N102K^ fibroblasts, PC were significantly longer than in control cells and failed to transduce SHH signaling, as demonstrated by absent *GLI1* and *PTCH1* upregulation following stimulation with the SHH agonist SAG (*Smoothened Agonist*) (Figure 2A,B). To further explore these defects, we examined ciliary composition and found marked reductions in ARL13B (25%), INPP5E (45%) and IFT88 (70%) (Figure 2C). ACIII and GPR161, two SHH‐related membrane proteins, were also decreased (30% and 47%, respectively) (Figure 2C).

We next investigated the basal body and TZ by classical IF and U‐ExM, which enables nanoscale mapping of TZ components [47]. Classical IF showed that TMEM17^N102K^ localizes to the TZ but at reduced intensity (~25%), consistent with Western blot results (Figures 1D and 2C). Other basal body and TZ proteins (centrin, γ‐Tubulin, CEP290, MKS1, NPHP4) localized normally, but TMEM67, a key TZ transmembrane component, was absent in patient cells, indicating that TMEM17 is required for its recruitment and proper TZ composition (Figure 2C). U‐ExM revealed that in control cells, TMEM17 forms a discontinuous concentric ring (251 ± 45 nm in diameter) that nearly overlaps with TMEM67 (255 ± 45 nm), while RPGRIP1L (217 ± 46 nm) lies closer to the axoneme. In terms of proximo‐distal positioning (axial localization relative to centrin), TMEM17 (100 ± 42 nm), TMEM67 (118 ± 4 nm) and NPHP4 (110 ± 26 nm) are closely localized and positioned above CEP290 (38 ± 28 nm) (Figure 2D–G, S1 Supplementary Table 3). In patient cells, RPGRIP1L showed altered localization: its axial position is significantly shifted from 130 ± 33 nm to 182 ± 22 nm, and its ring diameter decreased from 217 ± 46 nm to 117 ± 46 nm. Due to low TMEM17^N102K^ levels, its precise positioning in patient cells could not be determined (Figure 2E–G, S1 Supplementary Table 3). Together, these findings highlight TMEM17's essential role in TZ architecture: while not required for localization of NPHP4, CEP290, or MKS1, it is critical for TMEM67 recruitment and correct RPGRIP1L sub‐positioning.

### 3.3 Modeling in *C. elegans* of All *TMEM17* Variants Identified in Human Cases

To query the functional impact of TMEM17 patient variants, we used *C. elegans* as a complementary model after confirming that the affected residues, R94, G101, and N102, are conserved, corresponding to R73, G80, and N81 in the nematode ortholog (S1 Supplementary Figure 1).

### 3.4 The *C. elegans* TMEM‐17 Proteins Bearing Patient Variations: Correct TZ Localization but Defective Stability/Folding

**FIGURE 3:**
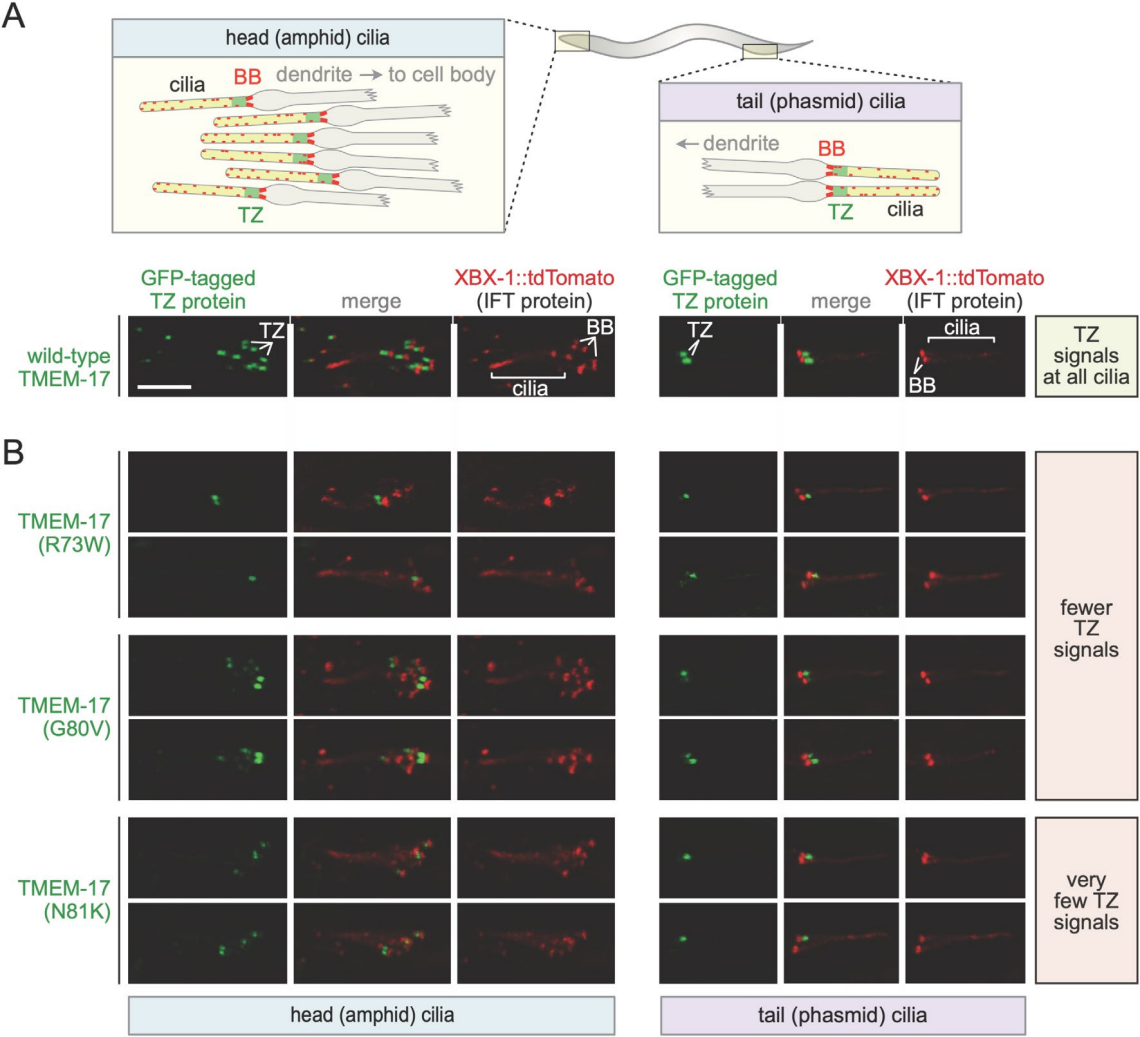
**Transition zone localization of** ***C. elegans*** **TMEM‐17 variants modeled on patient va riations is partially affected. (A)** Schematics show wild‐type localization of a TZ protein in the multiple head (including amphid) and two tail (phasmid) cilia. XBX‐1::Tdtomato docks at the basal body (BB) and travels as part of IFT particles on the ciliary axoneme (dots). Representative fluorescence images of GFP‐tagged, wild‐type TMEM‐17 (TMEM‐17::GFP) are shown below. **(B)** Localization patterns of GFP‐tagged TMEM‐17 variants (R73W, G80V and N81K) corresponding to patient variations. Fewer ciliated sensory neurons reveal the presence of the GFP‐tagged proteins at the TZ, and in cases where the proteins are observed at the TZ, their presence is reduced and less robust compared to the wild‐type protein.

To assess whether patient variants affect *C. elegans* TMEM‐17 stability or localization, we generated strains expressing GFP‐tagged wild‐type and mutant proteins under the endogenous promoter (S1 Supplementary Table 4). Wild‐type TMEM‐17 localized to the TZ of all ciliated neurons in the head and tail, forming pill‐shaped signals (Figure 3), similar to other TZ proteins [29]. In contrast, the R73W, G80V, and especially N81K variants localized to the TZ in fewer ciliated neurons, with localization of R73W and N81K particularly rare. Ultrastructurally, wild‐type TMEM‐17::GFP consistently showed 3–5 striations from discontinuous rings or spirals (S1 Supplementary Figure 3). Although fainter, similar patterns were seen for the variants, including a striking 4‐band image for G80V. These results suggest that a smaller proportion of each variant reaches the TZ, potentially indicating impaired stability/folding.

### 3.5 The *C. elegans* TMEM‐17 Proteins Bearing Patient Variations are Functionally Impaired

To test the functionality of *C. elegans* TMEM‐17 variants, we used CRISPR‐Cas9 to introduce point variations into the endogenous *tmem‐17* locus. Mutant strains (*tmem‐17[R73]*, *tmem‐17[G80], tmem‐17[N81]*) develop and move normally, consistent with cilia‐defective mutants, which display sensory defects but no major developmental or locomotion issues. TMEM‐17 loss does not influence the localization of MKS‐2 (ortholog of TMEM216), nor ciliary gate function [27, 44, 48, 49] (S1 Supplementary Figure 4), suggesting a more “peripheral” function compared to “core” TZ proteins in *C. elegans* [29]. Nevertheless, given its previously discovered cooperation with NPHP‐4 (ortholog of NPHP4) in ciliogenesis [44], we postulated that these variants might still influence TMEM‐17 function.

We previously uncovered that *tmem‐17* and *nphp‐4* genetically interact to regulate ciliogenesis [44]. Whereas single mutants exhibit minimal ciliogenesis defects, double mutants display severe structural abnormalities: transmission electron microscopy (TEM) reveals loss of TZ “Y‐links” and disrupted membrane anchoring, leading to prominent ciliary structure defects. This synergic phenotype reflects a known interaction between “MKS module” gene (e.g., *tmem‐17, mks‐1/2/3/6, or tmem‐237*) and “NPHP module” genes (*nphp‐4* or *nphp‐1*), leading to defective dye uptake by cilia exposed through the cuticle [50, 51, 52].

**FIGURE 4:**
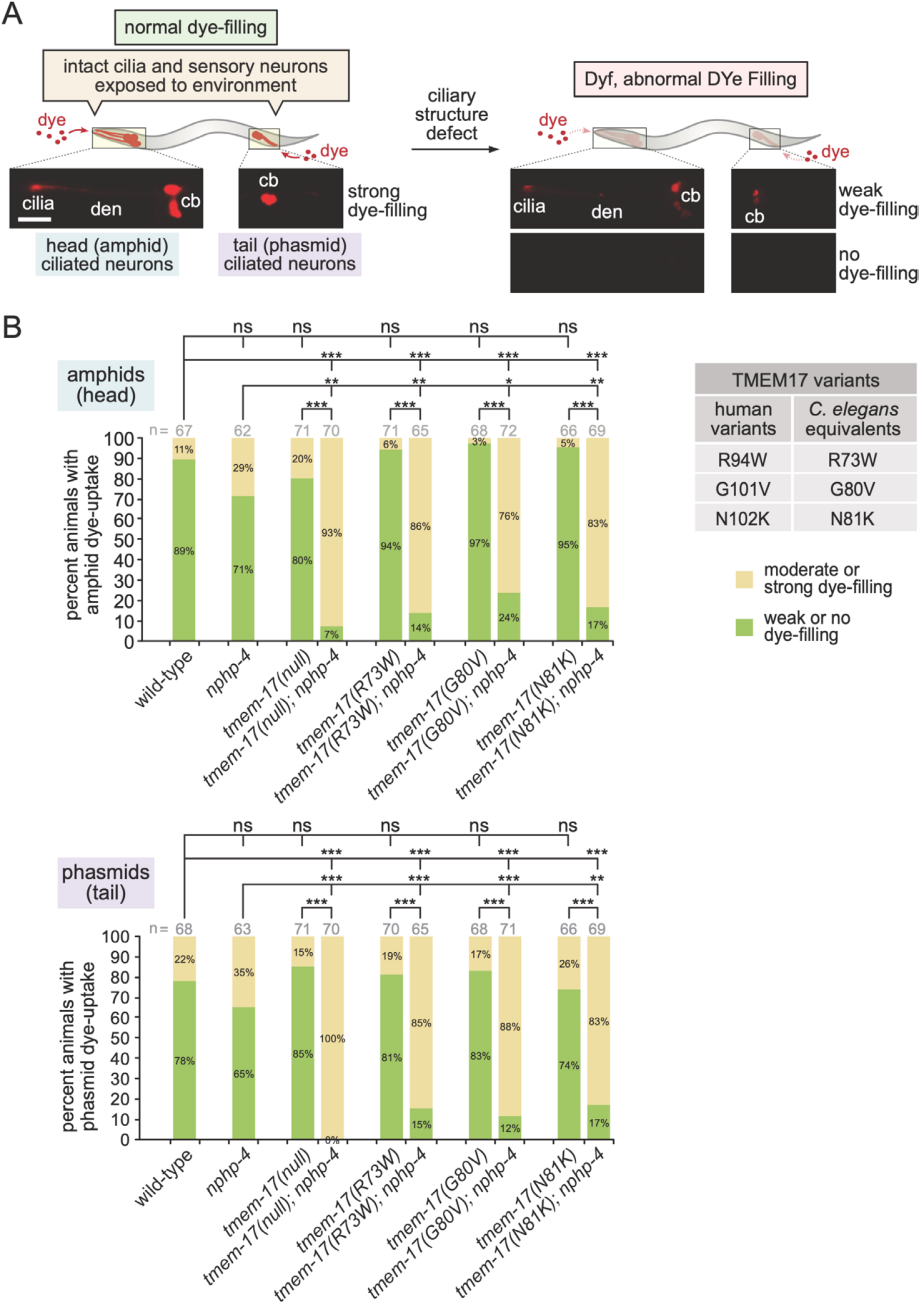
***C. elegans*** **TMEM‐17 variants modeled on patient variations are functionally impaired. (A)** Cilia at the distal ends of dendrites (den) in the amphids (head) and phasmids (tail) of *C. elegans* are normally exposed to the environment via pores in the cuticle. Exposure of cilia to DiI leads to uptake into the ciliated sensory neuron cell bodies (cb), as shown in the schematic and representative fluorescent images. Defects in ciliary structure (e.g., loss or shortening) causes an abnormal DYe Filling (DYF) phenotype characterized by weak or no detectable dye uptake. Scale bar, 40 μm. **(B)** Dye‐filling assays for wild‐type animals and the indicated mutant strains are shown for head and tail ciliated sensory neurons (top and bottom graphs, respectively). Dye uptake in all single mutants, namely *nphp‐4*, *tmem‐17(null)*, and three *tmem‐17* mutants bearing substitutions matching patient variants (R73W, G80V and N81K), is not statistically different from wild‐type. All *nphp‐4; tmem‐17* double mutant combinations are statistically different from wild type, as well as the *nphp‐4* and four *tmem‐17* single mutants. The prominent DYF phenotype of the double mutants involving the *tmem‐17* variants indicates functional defects comparable to the *tmem‐17* null mutant. ns, not significant; *p < 0.05; **p < 0.01; ***p < 0.001 (Tukey's HSD post hoc).

To assess ciliary structure, we therefore performed dye‐filling assays [50, 51, 52] (Figure 4A). Single mutants—*tmem‐17(null), tmem‐17* R73W, G80V, N81K, and *nphp‐4*—showed robust dye uptake, similar to wild‐type (Figure 4B). In contrast, double mutant combining *nphp‐4* with any *tmem‐17* mutants exhibits significant dye‐filling defects, affecting both head and tail cilia.

Together, our *C. elegans* data demonstrate that all three TMEM17 variants corresponding to MKS (Arg94Trp), OFD (Asn102Lys), and JBTS (Gly101Val) display reduced ciliary abundance and functions that are partially, if not fully, abrogated.

## 4 Discussion

TMEM17 is an established ciliary protein that functions at the TZ [29, 42, 44]. Despite its association with JS and OFD6 in two families [39, 41], it is not classified as a ciliopathy protein in the OMIM or CiliaMiner [53] databases. Here, we identified two MKS cases harboring the same founder homozygous *TMEM17* variant involving a highly conserved residue. Functional studies in human cells/tissues and *C. elegans* reveal that all three *TMEM17* missense variants impair protein stability and function, confirming *TMEM17* as a *bona fide* ciliopathy gene whose disruption can manifest in a phenotypic spectrum from OFD6/JS to fetal‐lethal MKS (S1 Supplementary Table 2). This aligns with the wide phenotypic spectrum in other TZ protein‐encoding genes and, more generally, in ciliopathy‐causing genes.

**FIGURE 5:**
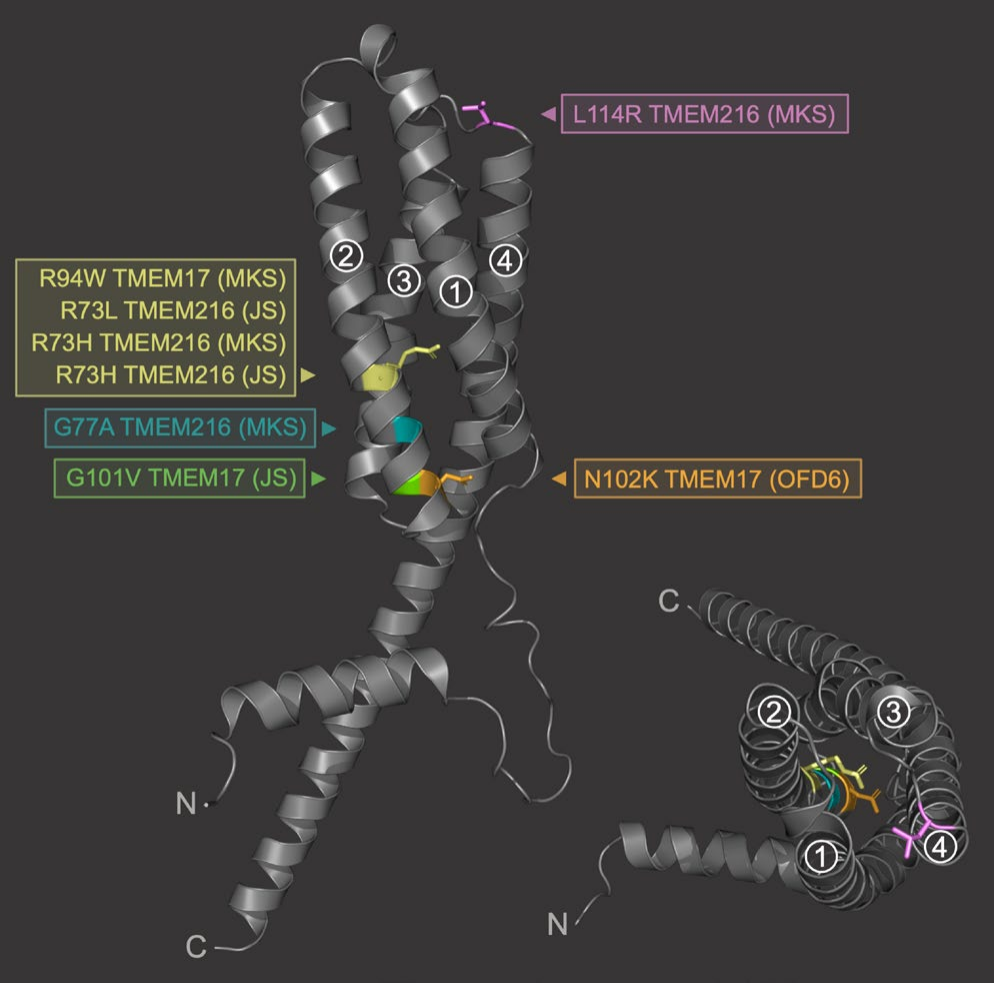
**Mutational hotspot within the predicted second helix of the related four‐transmembrane proteins TMEM17 and TMEM216.** Pymol cartoon representations (two orientations) of the Alphafold 2‐predicted TMEM17 protein structure, showing the positions of the patient variants from TMEM17 and related TMEM216 mapped onto the structure. The helices in the four‐transmembrane protein are marked by a number. Except for L114R, all patient variants cluster within the second transmembrane helix of the protein.

Remarkably, three unrelated families harbor pathogenic variations affecting the corresponding Arg73 residue in the related TMEM216 protein, also associated with JS (Arg73Leu, Arg73His and Arg 73Cys) and MKS (Arg73His) [35] (S1 Supplementary Figure 1). A nearby TMEM216 MKS variant (Gly77Ala) lies between the TMEM17 residues studied (Arg94 and Gly101/Asn102; S1 Supplementary Figure 1). In all, the six ciliopathy‐associated residues in TMEM17 and TMEM216 are among the most conserved (S1 Supplementary Figure 1), and in TMEM17 cluster within the second helix of the four‐transmembrane‐domain structure (Figure 5).

If residues within helix 2 are crucial for TMEM17 and TMEM216 function, what might be the pathomechanism(s) underlying their disruption?

First, our patient‐based studies argue all three identified *TMEM17* variants produce unstable proteins. Structural modeling suggests that these variants destabilize helix 2 by breaking a salt bridge (R94W), disrupting a hydrogen bond (N102K), or introducing a bulkier residue (G101V) (Figure 1D). Supporting this, kidney and lung tissues from case 1 (TMEM17^R94W^) show no detectable TMEM17. Additionally, TMEM17^N102K^ levels are largely reduced at the TZ in OFD6‐derived fibroblasts compared to control cells.

Second, IF analyses of TMEM17^R94W^ fetal kidney (case 1) and TMEM17^N102K^ cells (OFD6 case) reveal severe ciliogenesis defects, as previously suggested [39], with subsequent altered SHH signaling as demonstrated by assaying *GLI1* and *PTCH1* expression following SAG treatment. We demonstrate aberrant cilium elongation, altered recruitment of TZ protein TMEM67, and defective trafficking of key ciliary components, including membrane‐associated axonemal proteins (ARL13B, INPP5E, GPR161, SMO, and ACIII) and the IFT transport system protein IFT88. These findings align with previous analyses on *TMEM67−/−* RPE1 cells showing elongated cilia and mislocalized ARL13B and INPP5E [54]. The altered cilia length and composition result in impaired function, as TMEM17^N102K^ cells fail to transduce the SHH signaling. U‐ExM localized TMEM17 at the TZ forming nine clusters arranged in a ring whose diameter is consistent with membrane association, as shown for other transmembrane TZ‐proteins [55, 56]. In terms of proximal positioning, TMEM17 localizes at the same proximodistal level as TMEM67, RPGRIP1L, and NPHP4 but distinct from CEP290, which is more proximal to centrin (Figure 2). While the sub‐localization of CEP290 and NPHP4 in patient cells remains unchanged, RPGRIP1L appears more distal from the basal body (by 50 nm) and closer to the axoneme (by 100 nm). This is unexpected, considering the proposed role of RPGRIP1L as a scaffold protein. However, it may be relevant to our previous data in *C. elegans* , where MKS‐5 (RPGRIP1L orthologue) disperses distally along the axoneme in some TZ mutants (including *tmem‐17*), whereas other TZ proteins do not, suggesting that MKS‐5 does not necessarily co‐localize with other TZ proteins at the Y‐links [44]. Additional experiments in patient cells harboring variations in other TZ‐encoding genes will help clarify whether RPGRIP1L is part of the Y‐link assembly, acting as a scaffold or an assembly factor. By defining the TZ sub‐positioning of TMEM17 and its influence on other TZ proteins, we refine the functional hierarchy of TZ components.

Third, we used *C. elegans* as a complementary system to assess the impact of the TMEM17 variants. Unlike its human orthologue, the nematode TMEM‐17 protein has a more “peripheral” role in the assembly of the TZ (S1 Supplementary Figure 4). Using the known genetic interaction between *tmem‐17* and *nphp‐4 null* mutants [44], which cause ciliogenesis and dye‐filling defects, we tested the functionality of the *tmem‐17* variants. All three patient variants, like the *tmem‐17* null mutant, caused dye‐filling defects when combined with the *nphp‐4* mutant, revealing impaired ciliogenesis. The mechanism linking an “MKS module” protein (e.g., TMEM‐17) with an “NPHP module” protein (NPHP‐4) in TZ formation is unclear in *C. elegans* (also identified in mammals [57]), but may relate to synergistic roles for the TZ proteins in forming connections between the basal body and TZ regions to the overlying membrane [44]. Despite species differences in protein stability, assembly, and function, our collective data support that variants in TMEM1's second transmembrane helix disrupt its stability and function.

A major challenge in the field, especially for diagnosis and genetic counseling, is uncovering how variations in specific genes can result in such a wide phenotypic spectrum, often without clear genotype–phenotype correlation. *TMEM17* illustrated this well: all identified variants are missense and affect the same helix. Sometimes, simple mechanisms offer explanations, as exemplified in our study: complete loss of TMEM17 protein stability in MKS, despite the variant being missense, contrasts with the OFD6‐associated missense variant, which leads to only partial protein destabilization and may underlie the milder phenotype. Other mechanisms, such as basal exon skipping in *CEP290*, allow production of shortened but functional proteins [58]. However, the mechanisms are often poorly understood, as with our case, where two variations affecting the same transmembrane domain of TMEM17 are responsible either for isolated JS or JS associated with additional orofacial anomalies (OFD6). Functional interactions among TZ proteins occur, and functional interactions between TZ proteins and the IFT system (including Bardet‐Biedl syndrome (BBS)‐associated proteins) have also been documented in both humans and nematodes [29, 57, 59, 60]. Furthermore, beyond the allelism between MKS and BBS [61, 62], variations in three TZ protein‐encoding genes, *MKS1*, *TMEM63*, and *CEP290* may have a potential epistatic effect on variations in known BBS‐associated loci [62]. Hence, while ciliopathies are primarily inherited in a simple recessive manner, a more complex mode of inheritance involving genetic modifiers is currently proposed, placing these diseases at the boundary of simple and complex genetics [62, 63, 64]. Additional studies will continue to help illuminate the molecular etiology of distinct ciliopathies, which now includes *TMEM17* as a causative gene.

## Author Contributions

L.B., P.N., P.G., L.H., N.B., P.G., C.M., C.D., T.A.‐B., and S.T. performed genetics analyses. C.M., L.V., M.G.‐T., M.P., J.P., and S.T. handled human cell/tissue experiments. C.L. and D.A. worked on *C. elegans* . J.A. and P.G. performed autopsies. E.M.V. and V.S. provided the fibroblasts. T.A.‐B., M.R.L., and S.T. supervised the study. M.R.L. and S.T. wrote the manuscript with improvements from all authors.

## Ethics Statement

Medical terminations followed French and Argentinian laws with approval from our local committee. Autopsies and genetic testing were conducted after parental written consent. The study was approved by the CPP Paris Ile‐de‐France II (2009–164/DC‐2011‐1449) and the CEMIC Ethic Committee (IRB00001745–IORG0001315‐October 21, 2024).

## Conflicts of Interest

The authors declare no conflicts of interest.

## Supporting information


**S1:** Supplementary methods, figures and tables.

## Data Availability

The data that support the findings of this study are openly available in ClinVar at https://www.ncbi.nlm.nih.gov/clinvar/variation/3178950/?oq=SUB15250541&m=NM_198276.3(TMEM17):c.280C%3ET%20(p.Arg94Trp), reference number SUB15250541.
