# Supplementary material for: Missense Variants in the Second Transmembrane Domain of TMEM17 Disrupt Its Stability and Function and Lead to a Wide Phenotypic Spectrum of Ciliopathies
Source: Clin Genet. 2025 Aug 21;109(2):305–15. doi: 10.1111/cge.70042 (PMC12779253; doi:10.1111/cge.70042)
Supplement: Supplementary file 1 — S1: Supplementary methods, figures and tables. [file CGE-109-305-s001.docx]

**SUPPLEMENTARY MATERIAL**

**Supplementary Methods**

1. Autopsy
2. Trio exome sequencing
3. Cell culture
4. Immunofluorescence
5. Ultrastructure Expansion Microscopy
6. *C. elegans* strain creation and maintenance
7. *C. elegans* protein ciliary localization experiments
8. *C. elegans* dye-filling assays for assessing intact ciliary structures
9. Roaming and osmoavoidance (behavioral) assays

**Supplementary Figures**

1. *TMEM17* variants occur in evolutionarily conserved residues across TMEM proteins

of the transition zone.

1. CRISPR/CAS9-based generation of *C. elegans* *tmem-17* mutant strains.
2. Analysis of *C. elegans* mutated TMEM-17 localization at the ultrastructural level.
3. Disruption of *C. elegans* TMEM-17 does not impair transition zone gating or influence

the localization of MKS-2/TMEM-216.

1. Phenotypic analysis of roaming/exploration and osmoavoidance behaviors in *tmem*

*17* null and patient variant mutants.

1. Determination of the axial distance to centrin, ring diameter and lateral width of

TZ/TF proteins.

**Supplementary Tables**

1. Antibodies.
2. Clinical and molecular data of cases with *TMEM17* variations.
3. U-ExM analyses on control and OFD6 patient fibroblasts.
4. *C. elegans* strains generated.

**Supplementary References**

**Supplementary Methods**

1. **Autopsy**

According to the French and Argentinian laws, complete fetal autopsy was performed after parental consent and according to standardized protocols. Detailed examination included X-ray, photographs, macroscopic and histological examination of all viscera. Fetal biometric data were assessed according to the morphometric criteria of Guihard-Costa^1^. All tissue specimens were immediately fixed in 10% formalin solution and processed routinely for embedding in paraffin wax. They were stained with hematoxylin, eosin and safran (HES). Then the slides were examined by light microscopy. Fetal tissue samples were frozen at -80°C for molecular genetic tests with parental consent. Placenta was provided and examined after formalin fixation. For histological examination, blocks containing cord, membrane and full thickness of villous tissue were taken. Tissue sections of 3 μm thickness were performed on paraffin embedded biopsies and stained with HES.

1. **Trio exome sequencing**

Genomic DNA was extracted from fetal tissues (fetus 1 and fetus 2) or peripheral blood samples (parents). Agilent SureSelect libraries were prepared from 50ng of genomic DNA enzymatically fragmented (Agilent SureSelect Enzymatic Fragmentation Kit). Exome capture was performed with the 41 Mb Magnis SureSelect XT-HS kit, Human, All exon V8 (Family 1) or SureSelect Human All Exon V7 kit (Family 2) (Agilent technologies®). Sequencing was carried out on Illumina® sequencers (Family 1: NextSeq500-Illumina®, Necker-Enfants Malades-APHP Genomics Platform and Family 2: Macrogen Europe). Local bioinformatics pipelines were applied. For family 1 (Bioinformatic Platform, Université Paris-Cité, *Imagine* Institute, Dr P. Nitschké): After demultiplexing, alignment of paired-end sequences on human genome reference (NCBI build37/hg19 version) as well as variant calling were performed by the Illumina DRAGEN Bio-IT Platform version 4.2. An in-house software tool (PolyWeb: https://www.polyweb.fr/index.html) was used for annotation (based on Ensembl release 71) and filtration of variants according to relevant genetic models. For family 2: The mapping, alignment to genome reference (GRCH38) and variant calling procedure was carried out using the protocol developed in Bitgenia (Argentina), based on the good practices established by the Broad Institute (Eli and Edythe L. Broad Institute of Harvard and MIT). The Variant file (VCF) analysis was performed using B_platform software. We excluded known variants >1% in the public databases (GnomAD V2) as well as variants previously identified more than 30 times in “in-house” exomes. Variants were then classified according to ACMG recommendation^2^.

1. **Cell culture**

Fibroblasts were cultured in Opti-MEM (Gibco, 51985026) supplemented with 10% FBS (Gibco, 10270106) and 1% P/S (Gibco, 15140122). To induce ciliogenesis cells were starved in serum free medium (Opti-MEM with 1% P/S) for 48 hours. For immunofluorescence cells were plate on 24-well plates containing 12mm coverslips. For [SHH pathway](https://www-sciencedirect-com.proxy.insermbiblio.inist.fr/topics/biochemistry-genetics-and-molecular-biology/hedgehog-signaling-pathway) induction, smoothened [agonist](https://www-sciencedirect-com.proxy.insermbiblio.inist.fr/topics/biochemistry-genetics-and-molecular-biology/agonist) (SAG) (Santa Cruz Biotechnology, sc-202814) was used at a final concentration of 0.5µM in serum free medium for 24 hours, after 48 hours of starvation.

1. **Immunofluorescence**

Fibroblasts were fixed in ice-cold methanol for 5 min and then washed three times in PBS. Cells were permeabilized in 0.3% Triton X-100 (Sigma, X100) in PBS for 7 min, followed by a 1-hour incubation in blocking solution (1% BSA (Sigma, A2153), 10% goat serum (Sigma, G9023), 0.1% Tween20 (Sigma, P1379)). Cells were then incubated overnight at 4°C with primary antibodies (**Supplementary Table 1**) diluted in blocking solution. After three PBS washes, fibroblasts were incubated for 1 hour at room temperature in the dark with the appropriate secondary antibodies (1:500) in blocking solution. Finally, cells were washed 3 times and mounted on glass slides (Vectashield Vibrance, H-1700). Images were acquired using a Leica SP8 Confocal microscope (63x objective N.A 1.4) and analyzed with ImageJ software. The number of ciliated cells, primary cilium (PC) length, and protein intensity were quantified on maximum intensity Z-project. PC length was measured on the acetylated alpha tubulin (Ac-αTub) and gamma tubulin (γTub) channels by drawing a segmented line along the axoneme. To measure signal intensity for each transition zone (TZ) or PC protein of interest, the PC or TZ were segmented on the α-Tub and γ-Tub channels, and the average intensity was measured on the TZ/PC protein channel. For each image, the average intensity of five negative areas was measured and then subtracted from the protein value to eliminate background variability. Graphics represent mean± standard deviation (SD) and each point corresponds to one PC. Statistical analysis was performed with GraphPad Prism V10 software using statistical tests indicated in figure legends.

1. **Ultrastructure Expansion Microscopy**

We followed the U-ExM protocol described by Gambarotto et al., 2019^3^, with minor modifications. Control and patient cells cultured on 12 mm glass coverslips were fixed with methanol, then incubated in a freshly prepared solution of 2% acrylamide and 1.4% formaldehyde in PBS. After overnight incubation at 37°C, the coverslips were washed with PBS and placed cell-side down on a 35 μl drop of monomer solution (19% sodium acrylate, 10% acrylamide, 0.1% N,N'-Methylenebisacrylamide in PBS), to which 10% TEMED and 10% ammonium persulfate were added immediately before use. The coverslips were incubated for 5 min on ice, followed by 30 min at 37°C, then transferred to a denaturation buffer (200 mM SDS, 200 mM NaCl, 50 mM Tris-Base, pH 9) and agitated for 15 min to detach the gels. The gels were then cut into small pieces, incubated in denaturation buffer for 1.5 hours at 95°C, washed twice for 10 min in deionized water, and left overnight in water at 4°C to ensure full expansion. After 2 × 10 min washes in PBS, the gels were incubated overnight at 4°C with primary antibodies (**Suppl. Table 1**) diluted in saturation buffer (2% BSA, in PBS). The next day, the gels were washed 3 × 10 min in PBST (2% BSA, 0.1% Tween in PBS), incubated for 2 hours at 37°C with secondary antibodies, and washed again in PBST as previously described. Finally, the gels were incubated 2 × 30 min in deionized water, then left to expand overnight in deionized water to reach their maximum size. Expanded gels were mounted on 24 mm coverslips coated with poly-D-lysine (Gibco, A3890401) and imaged using a Nikon AX inverted confocal microscope equipped with a 60× oil immersion objective (Plan Apo, N.A. 1.42). 3D stacks were acquired with a 0.2 µm z-step and a 0.11 µm x, y pixel size. Finally, images were deconvolved in standard mode using Huygens software. To determine whether the variation impacts the architecture of the TZ, we measured the distances between different TZ and transition fiber (TF) proteins relative to centrin, comparing control and patient cells, as previously described^4^. Transversal images of TZ/TF protein signals obtained from U-ExM analyses using anti-TZ/TF protein antibodies, anti-GT335 (which marks polyglutamylated tubulin), and anti-centrin (which marks the basal body), were acquired simultaneously. Using Image J software, images were cleaned by a mean filter of 1 pixel, background subtracted and they were resized to 4096x4096 pixels before measurement. First, to determine the axial localization of TZ/TF proteins we measured the distance between the distal edge of centrin and the peak intensity of the TZ/TF protein of interest. On transversal images of the TZ, TZ/TF proteins are visible as two symmetrically distributed points on the axoneme. Thus, we were able to measure the lateral diameter representing the diameter of the ring and the total lateral width of TZ/TF proteins (more details in the **Suppl. Figure 6**). Graphics represent mean± standard deviation (SD) and each point corresponds to one primary cilium. Statistical analyses were performed with GraphPad Prism V10 software and statistical tests used are indicated in each figure legend. To illustrate the relative localization of different proteins of interest in the TZ, a seven-color super-resolution image was generated. This was achieved by superimposing 7 images of different TZ/TF proteins using ImageJ software. The images were cropped to a 40x40 pixel square, cleaned with a mean filter of 1 pixel, background-subtracted and they were resized to 80x80 pixels. The protein positions were adjusted based on the average axial distance obtained previously and the threshold was adjusted to represent their size.

1. ***C. elegans* strain creation and maintenance**

All *C. elegans* strains created and used in this study (listed in **Suppl. Table 3**) were maintained and cultured at 20ºC, as previously described ^5^. Standard genetic crosses were used to generate double mutants. Strains bearing mutations matching patient variants were introduced endogenously in the *tmem-17* locus using CRISPR-Cas9 engineering (created by SunyBiotech; sunybiotech.com). The mutant strains were outcrossed to wild-type 3X prior to analysis. Notably, the *tmem-17* (ZK418.3) gene structure shown in Wormbase (wormbase.org) is incorrect at the time of writing as it codes for a smaller TMEM-17 protein (145 amino acids) lacking conserved amino acids. The correct gene structure and predicted 186 amino acid protein is shown in **Suppl. Figure 2**, together with the positions of the *tmem-17* presumed *null* allele (*gk177766*; Q23**)* and patient mutations used in this study. GFP-fusion constructs expressing wild-type TMEM-17 or variants bearing mutations matching the human patient variants were created and verified by sequencing (*C. elegans* R73W, G80V and N81K correspond to human R94W, G101V and N102K, respectively). Transgenic animals expressing the constructs were made and selected for as previously reported^5^.

1. ***C. elegans* protein ciliary localization experiments**

Transgenic animals expressing the fluorescent reporters TRAM-1::tdTomato, MKS-2::GFP, XBX-1::tdTomato and TMEM-17::GFP (wild-type and R73W, G80V and N81K variants) were immobilized using 10 mM levamisole and imaged using an LSM880 laser scanning microscope with Airyscan (Zeiss). To enhance the spatial resolution of the fluorescent proteins within the TZ, we employed the Joint Deconvolution software package from Zeiss, which makes use of the 32 Airyscan detectors to resolve features down to ~90 nm. To gauge ciliary localization, at least 20 animals were imaged and analyzed using ZEN software.

1. ***C. elegans* dye-filling assays for assessing intact ciliary structures**

The ability of *C. elegans* to take up a fluorescent dye *via* intact, environmentally-exposed ciliary structures was assessed as previously described^6^. In brief, L4 larvae were incubated in Vybrant DiI (Invitrogen; 1:1,000-fold dilution of 1 mM stock in M9 buffer) for 30 min, allowed to roam on a plate seeded with bacteria for 1 hr to clear intestinal dye, and observed by fluorescence microscopy. Dye uptake into amphid (head) and phasmid (tail) sensory neurons was quantitated with in Velocity for over 60 animals per strain in triplicate.

1. **Roaming and osmoavoidance (behavioral) assays**

Roaming assay was performed as previously described^7^. Worms were synchronized to grow to day 1 adults after 3 days at 20°C. Standard 60 x 15 mm NGM plates were entirely coated with a thin layer of OP50. A single worm was transferred per plate, allowed to roam for 18 hours at 20°C, then removed. A roaming score was calculated by overlaying a 3 mm square grid on the treaded plate and counting the number of squares explored by the worm. At least 19 animals per strain were tested on 3 separate days. Glycerol avoidance (osmoavoidance) assay was performed as previously described ^7^. A 13 mm diameter ring of high osmolarity was created using 8 M glycerol on standard unseeded NGM plates. Subsequently 7-8 adult worms grown at 20°C were placed inside the ring. Worms were monitored for 10 minutes, and the proportion of animals able to escape the high osmotic barrier was measured. At least 168 animals per strain were tested on 5 separate days. For each assay, P-values were calculated using one-way ANOVA followed by a Tukey’s HSD post-hoc test.

**Suppl. Figure 1: *TMEM17* variants occur in evolutionarily conserved residues across TMEM proteins of the transition zone.** An amino acid sequence alignment of TMEM17 and two closely-related proteins (TMEM216 and TMEM80) across diverse ciliated eukaryotes reveals that the R94W, G101V and N102K TMEM17 patient variants affect highly conserved residues. Also mapped onto the alignment are known TMEM216 (MKS2) patient variants, two of which (R73H and R73L) corresponds to the R94 position in TMEM17. The association of the variants with different ciliopathies is indicated (MKS, Meckel syndrome; JS, Joubert syndrome; OFD, Oro-Facio-Digital syndrome). Identical residues are highlighted in varying shades of gray, depending on the number of species in which they are conserved. Residue numbers are shown on the left of the sequences.

**Suppl. Figure 2: CRISPR/CAS9-based generation of *C. elegans* *tmem-17* mutant strains. A:** The gene structure of *tmem-17* (ZK418.3) predicted by Wormbase (wormbase.org) is incorrect, as it lacks N-terminal and internal coding sequence that generate a smaller protein (145 amino acids) lacking conserved residues. The correct *tmem-17* spliced gene structure, which codes for a protein of 186 amino acids, is shown. The additional nucleotides and amino acids are shown in green. The *tmem-17* mutants generated (variants shown in red) are as follows: *gk177766* allele (Q23* likely null mutant) and three CRISPR-engineered variants (R73W, G80V and N81K). Only amino acid-changing variations are shown. **B:** All CRISPR-Cas9-generated *tmem-17* mutations are shown (both amino acid-changing and silent mutations).

**Suppl. Figure 3: Analysis of *C. elegans* mutated TMEM-17 localization at the ultrastructural level. A:** Schematic showing the internal organization of the TZ into rings (or potentially a spiral) formed by repeated units of Y-links (typically 4-5 but in some cases, fewer depending on cilium type) along its length. **B:** The enhanced resolving power provided by the Zeiss Airyscan Joint Deconvolution module (lateral resolution down to ~90 nm) reveals a sub-ciliary TZ arrangement of the fluorescent proteins consistent with the presence of multiple rings (or a spiral) within the TZ (enlarged in the insets). Scale bar, 4 µm. Compared to GFP-tagged wild-type TMEM-17, fewer TMEM-17 variants (R73W, G80V and N81K) can be observed at the TZ, although if present, their organization appears similar to wild-type.

**Suppl. Figure 4: Disruption of *C. elegans* TMEM-17 does not impair transition zone gating nor influence the localization of MKS-2/TMEM-216. A:** Schematic showing a cilium at the distal end of a dendrite. The periciliary membrane compartment (PCMC) is proximal to the TZ which is a ‘gate’ at the base of the ciliary compartment. **B:** All *tmem-17* mutants (null and three patient variants, as indicated) are not defective in their ability to restrict a protein, TRAM-1a, at the PCMC, an indication that the function of the TZ is intact. A control mutant lacking the core scaffolding protein CEP-290, which has severely abrogated TZ function, shows TRAM-1a mislocalizing (‘leaking’) into the ciliary compartment (asterisks). The *tmem-17* mutants also do not influence the localization of the ‘core’ MKS module protein MKS-2/TMEM-216, whereas the *cep-290* mutant exhibits mislocalization of MKS-2/TMEM-216 at the PCMC and within the cilium (dotted circle shows approximate position of TZ). den, dendrite. Scale bar, 4 µm.

**Suppl. Figure 5: Phenotypic analysis of roaming/exploration and osmoavoidance behaviors in *tmem-17* null and patient variant mutants.** **A:** Disruption of *C. elegans* *tmem-17* does not impair the roaming/exploration behavior of animals. Individual animals are allowed to roam for 18 hr, and number of tracks crossed are counted. *tmem-17;nphp-4* double mutants exhibit reduced roaming compared to the single *tmem-17* mutants, but this difference is not statistically significant compared to the *nphp-4* single mutant . The positive control strain (*mksr-2;nphp-4*) shows a synergistic defect compared to the *mks-2* or *nphp-4* single mutants. **B:** The *tmem-17(null);nphp-4* double mutant exhibits a synergistic osmoavoidance defect compared to the single mutants. The *tmem-17* variant combinations with *nphp-4* are not statistically significant from the *nphp-4* single mutant. In this assay, the proportion of animals able to escape the high glycerol osmotic barrier after 10 min is measured.

For each assay, statistical significance was calculated using one-way ANOVA followed by a Tukey’s HSD post-hoc test. n values denote the number of animals individually tested. ****, *p* < 0.0001.


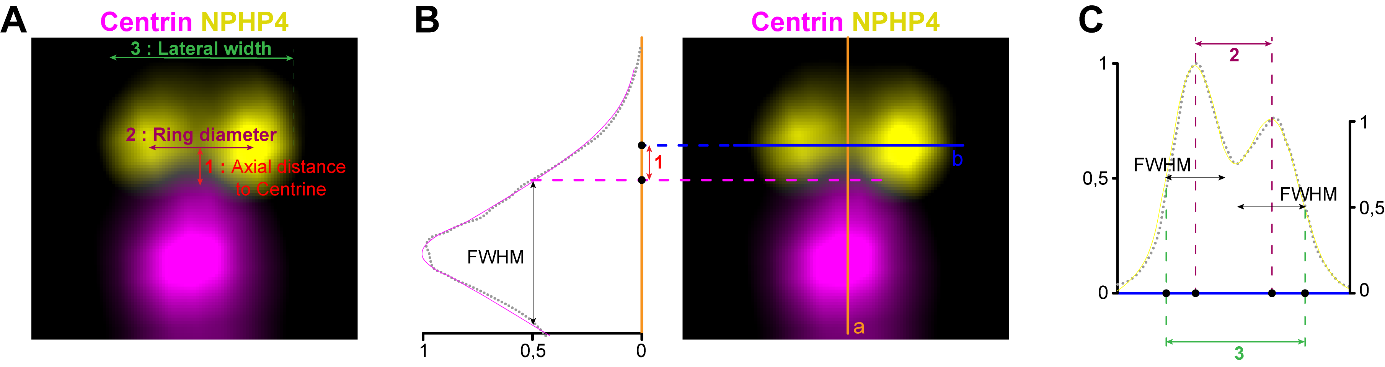


**Suppl. Figure 6: Determination of the axial distance to centrin, ring diameter and lateral width of TZ/TF proteins.** **A:** Schematic representation of the axial distance to centrin '1', ring diameter '2' and lateral width '3'. **B:** The axial distance of TZ/TF proteins to centrin was determined by calculating the distance between the distal edge of the centrin and the axis 'b' passing through the maximum intensity of the protein. The distal edge of centrin was defined as the position where the intensity profile of centrin, along axis 'a ', coincides with the full width at half maximum (FWHM). **C:** The ring diameter ’2’ was determined by measuring the peak to peak distance on the intensity profile along axis 'b' going through the maximum intensity of the protein. The lateral width '3' is represented by the distance between the outer edges of the two FWHM peaks.

**Supplementary Table 1: List of antibodies for immunofluorescence (IF), ultrastructure expansion microscopy (U-ExM) and Western Blot (WB) analyses.**

| Case | Clinical Diagnosis | CNS | Kidney | Liver | Polydactyly | Other | Nucleotide alteration (NM_198276.3) | Protein  change | Exon | AlphaMissense/REVEL/CADD scores | ACMG classification  (criteria) |
| --- | --- | --- | --- | --- | --- | --- | --- | --- | --- | --- | --- |
| 1 | MKS | OE | CK | BDP | Hands & feet | Shortened and curved long bones, posterior cleft palate | c.280C>T | Arg94Trp | 3 | 0.931 / 0.905 / 28.6 | class 4  (PS3, PM2, PP3) |
| 2 | MKS | OE, MIC | CK | HF | Hands & feet | spine curvature, lower limbs curved | c.280C>T | Arg94Trp | 3 | 0.931 / 0.905 / 28.6 | class 4  (PS3, PM2, PP3) |
| 3 | OFD6  (Li, 2016^5^) | MTS | - | - | Hands & feet | Orofacial anomalies | c.306C>A | p.Asn102Lys |  | 0.996 / 0.859 / 25.30 | class 4  (PS3, PM2, PP3) |
| 4 | JS  (Shamseldin, 2020^8^) | MTS | - | - | Hands & feet | Ptosis, PDA, thickened  pulmonary valve | c.302G>T | Gly101Val | 3 | 0.997 / 0.927 / 28.90 | class 4  (PS3, PM2, PP3) |

**Supplementary Table 2** **: Clinical and molecular data of cases with TMEM17 variations.** In addition to multiple lines of computational evidence, our functional analysis on human cells/tissues and *C. elegans*, support a deleterious effect of variants NM_198276.3: c.280C>T, c.306C>A and c.302G>T on TMEM17 and suggest their classification as **Class 4** (likely pathogenic) according to ACMG guidelines. CNS : central nervous system, OE: occipital encephalocele, MIC : microcephaly, CK : cystic kidneys, BDP : bile duct proliferation of liver, HF : hepatic fibrosis, MTS : molar tooth sign, MKS: Meckel syndrome, OFD6 : Orofaciodigital syndrome type 6, JS : Joubert syndrome

**Supplementary Table 3: U-ExM analyses on control and OFD6 patient fibroblasts.** Determination of axial positions of TF/TZ proteins using centrin as a reference coordinate (axial distance to centrin), radial positions by measuring ring diameter (peak to peak distance) and lateral width.

­ **Supplementary Table 4: *C. elegans* generated and used in this study.**

**Supplementary References**

1. Guihard-Costa AM, Ménez F, Delezoide AL. Organ weights in human fetuses after formalin fixation: standards by gestational age and body weight. *Pediatr Dev Pathol*. 2002;5(6):559-578. doi:10.1007/s10024-002-0036-7

2. Richards S, Aziz N, Bale S, et al. Standards and guidelines for the interpretation of sequence variants: a joint consensus recommendation of the American College of Medical Genetics and Genomics and the Association for Molecular Pathology. *Genet Med*. 2015;17(5):405-424. doi:10.1038/gim.2015.30

3. Gambarotto D, Zwettler FU, Le Guennec M, et al. Imaging cellular ultrastructures using expansion microscopy (U-ExM). *Nat Methods*. 2019;16(1):71-74. doi:10.1038/s41592-018-0238-1

4. Yang TT, Su J, Wang WJ, et al. Superresolution Pattern Recognition Reveals the Architectural Map of the Ciliary Transition Zone. *Sci Rep*. 2015;5:14096. doi:10.1038/srep14096

5. Li C, Jensen VL, Park K, et al. MKS5 and CEP290 Dependent Assembly Pathway of the Ciliary Transition Zone. *PLoS Biol*. 2016;14(3):e1002416. doi:10.1371/journal.pbio.1002416

6. Williams CL, Li C, Kida K, et al. MKS and NPHP modules cooperate to establish basal body/transition zone membrane associations and ciliary gate function during ciliogenesis. *J Cell Biol*. 2011;192(6):1023-1041. doi:10.1083/jcb.201012116

7. Sanders, A. A. W. M, Kennedy, J, Blacque, O. E. Image analysis of Caenorhabditis elegans ciliary transition zone structure, ultrastructure, molecular composition, and function. In: *Methods in Cell Biology*. Vol 127. Basto, R. & Marshall, W. F. Academic Press; 2015:323-347.

8. Shamseldin HE, Shaheen R, Ewida N, et al. The morbid genome of ciliopathies: an update. *Genet Med*. 2020;22(6):1051-1060. doi:10.1038/s41436-020-0761-1
